# Hyperglycemia and Loss of Redox Homeostasis in COVID-19 Patients

**DOI:** 10.3390/cells11060932

**Published:** 2022-03-09

**Authors:** María Elena Soto, Verónica Guarner-Lans, Eulises Díaz-Díaz, Linaloe Manzano-Pech, Adrían Palacios-Chavarría, Rafael Ricardo Valdez-Vázquez, Alfredo Aisa-Álvarez, Huitzilihuitl Saucedo-Orozco, Israel Pérez-Torres

**Affiliations:** 1 Department of Immunology, Instituto Nacional de Cardiología Ignacio Chávez, Juan Badiano 1, Sección XVI, Tlalpan, Mexico City 14080, Mexico; elena.soto@cardiologia.org.mx (M.E.S.); alfredoaisaa@gmail.com (A.A.-Á.); huitzilihuitls@hotmail.com (H.S.-O.); 2 Department of Physiology, Instituto Nacional de Cardiología Ignacio Chávez, Juan Badiano 1, Sección XVI, Tlalpan, Mexico City 14080, Mexico; veronica.guarner@cardiologia.org.mx; 3 Department of Reproductive Biology, Instituto Nacional de Ciencias Médicas y Nutrición Salvador Zubirán, Vasco de Quiroga 15, Sección XVI, Tlalpan, Mexico City 14000, Mexico; eulisesd@yahoo.com; 4 Department of Cardiovascular Biomedicine, Instituto Nacional de Cardiología Ignacio Chávez, Mexico City 14080, Mexico; loe_mana@hotmail.com; 5 Critical Care Unit of the Temporal COVID-19 Unit, Citibanamex Center, Mexico City 11200, Mexico; a2novi@hotmail.com (A.P.-C.); rrvaldezvazquez@gmail.com (R.R.V.-V.)

**Keywords:** SARS-CoV-2, homeostasis redox, hyperglycemia, lipid peroxidation, selenium, thiols, nitrotyrosine, H_2_S

## Abstract

The infection with SARS-CoV-2 impairs the glucose–insulin axis and this contributes to oxidative (OS) and nitrosative (NSS) stress. Here, we evaluated changes in glucose metabolism that could promote the loss of redox homeostasis in COVID-19 patients. This was comparative cohort and analytical study that compared COVID-19 patients and healthy subjects. The study population consisted of 61 COVID-19 patients with and without comorbidities and 25 healthy subjects (HS). In all subjects the plasma glucose, insulin, 8-isoprostane, Vitamin D, H_2_S and 3-nitrotyrosine were determined by ELISA. The nitrites (NO_2_^−^), lipid-peroxidation (LPO), total-antioxidant-capacity (TAC), thiols, glutathione (GSH) and selenium (Se) were determined by spectrophotometry. The glucose, insulin and HOMA-IR (*p* < 0.001), 8-isoprostanes, 3-nitrotyrosine (*p* < 0.001) and LPO were increased (*p* = 0.02) while Vitamin D (*p* = 0.01), H_2_S, thiols, TAC, GSH and Se (*p* < 0.001) decreased in COVID-19 patients in comparison to HS. The SARS-CoV-2 infection resulted in alterations in the glucose–insulin axis that led to hyperglycemia, hyperinsulinemia and IR in patients with and without comorbidities. These alterations increase OS and NSS reflected in increases or decreases in some oxidative markers in plasma with major impact or fatal consequences in patients that course with metabolic syndrome. Moreover, subjects without comorbidities could have long-term alterations in the redox homeostasis after infection.

## 1. Introduction

Coronavirus disease 2019 (COVID-19) is caused by the type 2 β-coronavirus and it induces a severe acute respiratory syndrome (SARS-CoV-2). This disease causes multiple organ failures that results from an exacerbated cytokine storm by the immune system. The cytokine storm is initially aimed at bringing down the infection and may have as a consequence, a possible fatal outcome for the patient [1].

COVID-19 impairs glucose homeostasis and metabolism in non- and diabetes mellitus (DM) and metabolic syndrome (MS) patients due to the cytokine storm, inflammation, angiotensin II-converting enzyme (ACE2) down-regulation and the direct injury to the pancreatic β-cells [2]. It also deteriorates the already impaired glucose homeostasis in patients with DM and in patients with other comorbidities such as obesity, insulin resistance (IR), hyperinsulinemia and hypertension in whom alterations in insulin secretion are already present and contribute to hyperglycemia [3]. Furthermore, increased inflammation and massive production of cytokines generate IR which adds to the deterioration of the insulin secretion by β-cells [4].

In addition, high glucose levels also contribute to the virulence and replication of SARS-CoV-2 since it is associated with a decrease in the number of phagocytic and nuclear polymorphic leukocytes [4]. Moreover, the virus sequesters the host’s mitochondrial function and shifts it from aerobic to anaerobic [5]. In this state, the pyruvate produced from glucose during glycolysis is oxidized to lactate, thereby increasing glucose levels in the cytosol and leading to the generation of limited amounts of adenosine-5-triphosphate (ATP) [5,6]. The viral replication also consumes large amounts of ATP and therefore, its concentration is depleted. In this condition, lactate is not metabolized by gluconeogenesis and accumulates in the blood, leading to a loss of the balance in glucose metabolism [7]. The increase in hyperglycemia contributes a high production of inflammatory cytokines and cellular mediators implied in pro-thrombotic process present in COVID-19 patients. [8]. All these alterations favor oxidative stress (OS) but also, the viral infection induces the cytokine storm by the immune system that contribute to OS [9]. This alteration leads to positive feedback systems for each element and/or metabolic pathway and may contribute to severe pneumonia with possible multiple organ failure (MOF) in the COVID-19 patient.

On the other hand, an overproduction of reactive oxygen species (ROS) leads to a deprivation of the antioxidant mechanisms which are crucial to decrease or abolish viral replication and the level of infection present in the patient [10]. Therefore, SARS-CoV-2 favors an increased threshold in the production of ROS and reactive nitrogen species (RNS). The overproduction of these molecules is associated with elevated activity and/or expression of the inducible nitric oxide synthase (iNOS), nicotinamide-adenine dinucleotide phosphate (NADP) oxidases, cyclooxygenase 2, xanthine oxidase and with alterations in the mitochondrial functions that activate transcription factors such as nuclear factor kappa B subunit (NFkB) resulting in an exacerbated proinflammatory state and the production of interleukins [5,11]. Therefore, Interleukin (IL)-6, IL-8 and tumor necrosis factor alpha (TNF-α) are increased in bronchial epithelial cells and alveolar macrophages and these cytokines can then activate macrophages and neutrophils, resulting in the destruction of the alveolar wall, the collapse of small airways, hyper-permeability of pulmonary capillaries and pulmonary edema, that result in the deterioration of pulmonary gas exchange [10]. These changes contribute to acute respiratory distress syndrome (ARDS), chronic obstructive pulmonary disease (COPD) and acute lung injury (ALI) [1]. However, the complex combination of the alterations in glucose metabolism and the association with the loss of redox homeostasis in COVID-19 patients have not been completely analyzed. Therefore, the aim of this study was to evaluate changes in glucose metabolism that could contribute to the loss of redox homeostasis in the plasma of patients with moderate and severe pneumonia by SARS-CoV-2 with and without comorbidities. In this study, we also tested whether the use of the presence of the anti-N protein and anti-S protein antibodies of the SARS-CoV-2 virus at the time of admission of the patients can be used as a diagnostic tool for COVID-19.

## 2. Materials and Methods

### 2.1. Study Type

This was a comparative cohort and analytical study that compared COVID-19 patients and healthy subjects (HS). The study was run between 20 August and 20 September 2020. The study population consisted of 61 patients over 18 years of age who were admitted to the intensive care unit (ICU) of the CITIBANAMEX Center and that had not developed septic shock, secondary to moderate or severe pneumonia due to COVID-19. Diagnostic criteria for septic shock were based on the Sepsis-3 consensus [12]. Exclusion from this study occurred when patients were younger than 18 years of age, when they were not able to grant an informed consent or when they refused to be included. Patients were also excluded if pregnant or breastfeeding or if they were under chronic use (last 6 months) or recent use of steroids, statins or antioxidants. The hospitalized patients included were considered to have moderate or severe symptoms considering their ventilatory status. Moderate COVID-19 patients were classified according to the Horowitz index which is defined as the ratio of partial pressure of oxygen (PaO_2_ in mmHg) in blood to the fraction of oxygen in inhaled air (FiO_2_), (PaO_2_/FiO_2_) with value index percentage of fraction >200 mmHg [13]. Patients with the severe condition required invasive mechanical intubation according to the criteria of Berlin for ARDS. The Berlin definition proposes 3 categories of ARDS based on the severity of hypoxemia: mild (200 mmHg < PaO_2_/Fio2 ≤ 300 mmHg), moderate (100 mmHg < PaO_2_/FiO_2_ 200 mmHg) and severe (PaO_2_/FiO_2_ ≤ 100 mmHg), along with explicit criteria related to timing of the syndrome’s onset, origin of edema and the chest radiographic findings. The ARDS definition task force was considered [14].

Twenty-five HS were matched by age and gender. HS were negative for SARS-CoV-2. The collection of peripheral blood samples was carried out by venipuncture. In these subjects, there was no suspicion of inflammatory disease or presence of degenerative disorders such as thyroid and autoimmune diseases, DM, dyslipidemia, arterial hypertension and MS. The intake of some medications that could interfere with the results of the study such as antioxidant drugs and non-steroidal anti-inflammatory drugs was considered, and the drugs were suspended 48 h before the obtainment of the sample. Ethical approval to perform the study was obtained from the local ethics committee on 19 August 2020 (Control-9867/2020, register REG. CONBIOETICA-09-CEI-011-20160627). A written informed consent for enrollment or consent to use data from the patients was obtained directly from them or their legal surrogates. The protocol was registered (TRIAL REGISTRATION: ClinicalTrials.gov (accessed on 6 February 2022) Identifier: NCT 04570254). Results related to this study were previously reported by Chavarría et al. during the pre-treatment and post-treatment evaluation with antioxidants [11]. We observed that the glucose levels were increased since the admission of the patients. It was therefore considered that, in addition to the main objective, the evaluation if this increase needed to be explored since it could be related to the oxidative background present in these patients.

The collection of peripheral blood samples was carried out by venipuncture when patients entered the ICU and they tested positive by the qRT-PCR test. In total, 34 moderate and 27 severe COVID-19 patients were included and 25 HS. The blood samples were centrifuged for 20 min at 936 g and 4 °C. The plasma of the samples was placed in 3 or 4 aliquots and stored at −30 °C.

### 2.2. Collection of Samples to Verify Infection by SARS-CoV-2 upon Admission to the Hospital

Paired saliva and nasopharyngeal swab samples were collected from all patients who were suspected to be infected by SARS-CoV-2. Samples were classified as positive for SARS-CoV-2 when both the N1 and N2 primer-probe sets were detected. The presence of the SARS-CoV-2 virus was evaluated using specific probes for the detection of the virus in conjunction with the real-time reverse transcriptase polymerase chain reaction technique (qRT-PCR). To evaluate organ dysfunction, the SOFA score (neurologic, respiratory, hemodynamic, hepatic and hematologic) was calculated at admission [12].

### 2.3. Detection in Plasma of the N and S Protein Antibodies of the SARS-CoV-2 Virus

The anti-N and anti-S protein antibodies of the SARS-CoV-2 virus were detected using two ELISA-type immunoassays. The plates were coated with 100 µL/well of 5 µg/mL of recombinant SARS-CoV-2 nucleocapsid protein derived from *E. coli* (CODE: 230-01104) or 2.5 µg/mL of recombinant SARS-CoV-2 Spike protein, S1 subunit derived from *E. coli* (CODE: 230-01101), supplied by RayBiotech Company (RayBiotech Inc., Georgia, GA, USA) and 100 µL per well of diluted plasma of the patients and added to the plates. The absorbance in the plates was measured on an ELISA reader Chromate 4300 (Awareness Technology, Inc., Palm City, FL, USA), at 450 nm. The percentages of positivity of each patient, for each type of anti-viral protein antibodies were evaluated and calculated using the following mathematical model: [(Absorbance at 450 nm of the patient/absorbance at 450 nm of the negative control) − 1] × 100, for both anti-N protein and anti-S protein antibodies [15].

### 2.4. Laboratory Tests

Laboratory tests were made for the COVID-19 patients to determine acute-phase reactants, hemoglobin, leukocytes, lymphocytes, platelets, creatinine, urea nitrogen, glucose, C-reactive protein (CRP), albumin, D-dimer, ferritin, IL-6 and oxygen saturation. Data from the patient’s medical history including demographic data, prior illnesses to infection by SARS-CoV-2, test result for COVID-19 and whether mechanical ventilation were used for the analysis of the results.

### 2.5. Glucose, Insulin, and HOMA-IR Concentrations

Glucose concentrations were determined using the enzymatic commercial kit from Pointe Scientific (Pointe Scientific Inc., Michigan, MO, USA), and insulin was determined by radioimmunoassay as previously described by Jiménez et al. [12]. (The HOMA index of IR was calculated by HOMA−IR = (insulin μU/mL × glucose mmol/L)/22.5 [16].

### 2.6. 8-Isoprostane, Vitamin D, H_2_S, and 3-Nitrotyrosine Concentrations

The kits for the determination of 8-isoprostane and Vitamin D were provided by Cayman Chemical Company, Michigan MO, USA. (8-isoprostane ELISA kit Item No. 516351 and vitamin D ELISA kit Item No. 501050). This assay is based on the competition between 8-isoprostane and an 8-isoprostane–acetycholinesterase conjugate for a limited number of 8-isoprostane-specific rabbit antiserum binding sites. The product of this enzymatic reaction has a distinct yellow color and adsorbs strongly at 412 nm. The assay for Vitamin D is based on the competition between vitamin D and a conjugated vitamin D-acetyl-cholinesterase. The product of this enzymatic reaction has a distinctive yellow color and absorbs strongly at 412 nm. Hydrogen sulfide (H_2_S) was quantified by a commercial colorimetric assay kit obtained from Elab science Biotechnology Co., Ltd., Houston, TX, USA. (Cat No. E-BC-K355-M). H_2_S reacts with an acetate solution to form ZnS which can be dissolved in an alkaline solution. Methylene blue is formed in the presence of Fe^3+^ and can absorb at 665 nm. 3-nitrotyrosine (3-NT) was determined with a kit provided by LifeSpan BioSciencies, Seattle, WA, USA) (3-nitrotyrosine ELISA kit No. LS-40120). This assay is based on the competitive ELISA principle and is measured at a wavelength of 450 nm. The measurements were made using a visible light micro plate reader (Stat Fax 3200 Awareness Technology Palm City, FL, USA).

### 2.7. Oxidative Stress Markers

#### 2.7.1. Nitrites (NO_2_^–^)

The NO_2_^–^ levels in plasma were determined by the Griess reaction. In total, 100 μL of plasma previously deproteinated with 0.5 N, NaOH and 10%, ZnSO_4_ were centrifuged at 1789× *g* for10 min. The supernatant was recovered and 200 µL of 1% sulfanilamide and 200 µL of 0.1% N-naphthyl-ethyl diamine were added. The total volume was adjusted to 1 mL. The calibration curve was obtained with a solution of KNO_2_ 5–0.156 nM and the absorbance was measured at 540 nm [17].

#### 2.7.2. Lipid Peroxidation Levels (LPO)

In total, 50 µL CH_3_-OH with 4% BHT plus phosphate buffer pH 7.4 were added to 100 µL of plasma. The reaction tube was incubated to 100 °C for 1 hour and centrifuged at 936 g at room temperature for 2 min. Then, the n-butanol phase was extracted, and the absorbance was measured at 532 nm [18]. This test is based on the reaction of malondialdehyde, a secondary product of the oxidation of fatty acids with three or more bonds, with thiobarbituric acid in an acid medium and at high temperature, generating a pink-colored product, the value is expressed in nM of malondialdehyde (MDA) per 1 mL of plasma.

#### 2.7.3. Evaluation of Total Antioxidant Capacity (TAC)

In total, 100 μL of plasma were suspended in 1.5 mL of a reaction mixture prepared as follows: 300 mM acetate buffer pH 3.6, 20 mM FeCl_3_ 6H_2_O and 10 mM of 2,4,6-Tris-2- pyridyl-s-triazine dissolved in 40 mM HCl. These reactants were added in a relation of 10:1:1 *v*/*v*, respectively. After mixing, the samples were incubated at 37 °C for 15 min in the dark. The absorbance was measured at 593 nm [19].

#### 2.7.4. Thiol Concentrations

The technique used was previously described by Erel and Neselioglu [20], with some adaptations and modifications carried out in our laboratory. In total, 50 µL of plasma were suspended reduced with 100 µL of KBH_4_ 10 mM dissolved in CH_3_OH-bidistilled H_2_O, (1:1 vol/vol) for 3 min, then, 700 µL of buffer (6.7 mM formaldehyde, 10 mM EDTA and Tris 100 mM, pH 8.2) was added for 3 min. Then, 100 µL of DTNB 10 mM in CH_3_OH was added for 4 min. The calibration curve was obtained with solution GSSG 1 mg/1 mL and the absorbance was measured at 415 nm.

#### 2.7.5. Glutathione Levels (GSH)

In total, 100 μL of plasma previously deproteinized with 20% trichloroacetic acid (vol/vol) and centrifugated to 10,000× *g* for 5 min plasma was added to 800 μL of phosphate buffer 50 mM, pH 7.3, plus 100 μL of 5, 5′-dithiobis-2-nitrobenzoic acid 1 M. The mixture was incubated at room temperature for 5 min and absorbance was read at 412 nm [18].

#### 2.7.6. Selenium

The technique used was previously described by Soto et al. [21]. All solutions were made with tridistilled H_2_O and were only used for the assay and discarded. In brief, in new Corning sterile polypropylene centrifuge tubes, 200 µL of plasma and 500 µL of acid mixture (4:1 vol/vol of HNO_3_ + HCl) plus 500 µL of 10% H_2_O_2_ were added and incubated at 120 °C for 4 h. After incubation, 100 µL of tridistilled H_2_O, 150 µL of 0.5 N NaOH, 200 µL of 30% formaldehyde, 200 µL of a mixture containing 0.5 N of N_2_S and 0.5 N of Na_2_SO_3_, plus 250 µL of 0.01 M of EDTA (pH 10.2), and 300 µL of 4 mM of toluidine blue were added. Samples were incubated for 15 min at 25 °C. At the end of the incubation, they were centrifuged at 448 rcf for 2 min and the absorbance was read at 600 nm. The calibration curve was performed using 100 ng/mL Na_2_SeO_3,_ and the samples were treated under conditions similar to those of the experimental samples.

### 2.8. Statistical Analysis

The Sigma Plot 14 program (Jendel Corporation, San José, CA, USA, 1986–2017) was used to generate the analysis and graphs. Statistical significance was determined by the Mann–Whitney rank sum test followed by the normality test (Shapiro–Wilk). Differences were considered statistically significant when *p* ≤ 0.05. The calculation of the antibodies against N and S proteins of the SARS-CoV-2 virus was carried out by the analysis of frequency performed using Fisher’s exact test.

## 3. Results

### 3.1. Demographic Characteristics

A total of 61 COVID-19 patients were examined, out of which 44 (72%) were men and 17 (28%) were women. Patients had an age range of 56 ± 13 years. The average body mass index was 29 ± 4 kg/m^2^. Normal weight was found in 13 (21%) and overweight in 24 (39%). Comorbid conditions prior to SARS-CoV-2 infection were dyslipidemia in 11 (18%), systemic arterial hypertension (SAH) in 7 (8%), DM in 6 (10%), DM plus dyslipidemia in 5 (8%), DM plus SAH in 5 (8%), SAH plus dyslipidemia in 3 (5%), SM in 9 (15%), chronic obstructive lung disease in 1 (1.6%) and chronic kidney disease in 2 (3.3%). Temperature was 36.6 ± 0.46 °C. Other variables expressed as median and minimum–maximum ranges, respectively, are included such as arterial blood oxygen pressure (PaO_2_, 66.9, 34–223 mmHg), partial pressure of carbon dioxide (PCO_2_, 31.7, 12.2–81.2 mmHg), Kirby’s index which is PaCO_2_/inspired fraction of oxygen (FiO_2_ 128, 26.8–299 mmHg), oxygen saturation (SpO_2_/FiO_2_ 138, 50–280 mmHg), urea (16, 5.6–106.7 mg/dL), ureic nitrogen (16, 5.6–196.7 mg/dL), total cholesterol (136, 69–217 mg/dL), triglycerides (133, 62–726 mg/dL), high-density lipoprotein (31, 14–60 mg/dL), low-density lipoprotein (70, 28–40 mg/dL), total bilirubin (0.60, 0.12–4.10 mg/dL), direct bilirubin (0.20, 10–1.20 mg/dL), leukocytes (8.8, 2–25 10^3^/μL), lymphocytes 0.8, 0.14–9.6 10^3^/μL), platelets median (244, 16–576 10^3^/μL), ferritin 541, 147–2592 ng/mL, CRP 146, 20–2450 mg/L), IL-6 67, 7.8–638.5 pg/mL) and D-dimer 700, 136–16,400 μg/mL). The demographic characteristics of the COVID-19 patients are shown in the Table 1.

### 3.2. Detection of Antibodies against the N and S Proteins of the SARS-CoV-2 Virus

The presence of anti-N protein and anti-S protein antibodies of the SARS-CoV-2 virus was determined in the present study at the time of admission. In the baseline measurement, 53 of the 61 patients tested positive for the presence of anti-N protein antibodies (86.9%), while 8 patients, 4 corresponding to the group who had a moderate respiratory disease, and 4 corresponding to the group of patients who progressed poorly and required the use of an artificial respirator, showed an absence of anti-N protein antibodies. A week later, seven of the eight patients who did not have detectable anti-N protein antibodies at the beginning, already tested positive for the presence of this type of antibody. The frequencies of positivity of the antibodies against the N and S proteins were of 31/34 with 91.2% frequency and 7/34 with 20.6% frequency, respectively, in patients with a moderate illness and of 26/27 with 96.3% frequency and 7/27 with 25.9% frequency, respectively, in patients with severe COVID-19. There was no statistical difference between the groups with moderate or severe acute respiratory disease in the analyses of frequency performed using Fisher’s exact test. Therefore, the results suggest that the serological response to the SARS-CoV-2 virus does not appear to be the condition responsible for progression to a more severe stage requiring artificial ventilation.

### 3.3. Glucose, Insulin and HOMA-IR Concentrations

**Figure 1:**
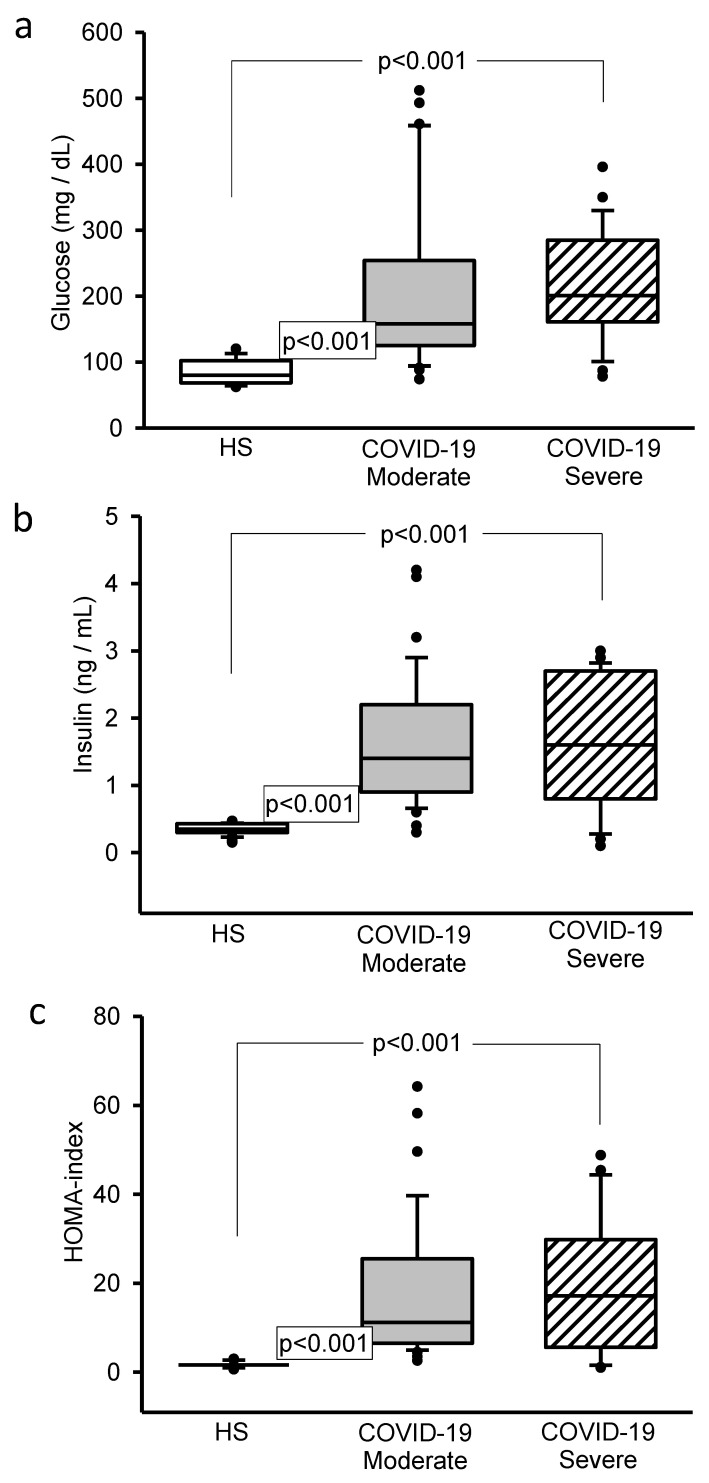
(**a**) The values of glucose significantly increased in the moderate and severe COVID-19 patients in comparison with HS. (**b**) Insulin had a significant increase in the moderate and severe COVID-19 patients in comparison to HS. (**c**) The HOMA index significantly increased in the moderate and severe COVID-19 patients in comparison with HS. Abbreviations: HS = healthy subjects, HOMA index = marker of insulin resistance which if the values are greater than 2.5 is an indication of insulin resistance.

The moderate and severe COVID-19 patients showed a significant increase (*p* < 0.001) in glucose, insulin concentrations and HOMA-IR in plasma in comparison with the HS, (Figure 1a–c, respectively).

### 3.4. 8-Isoprostane, Vitamin D, H_2_S, and 3-Nitrotyrosine Concentrations

**Figure 2:**
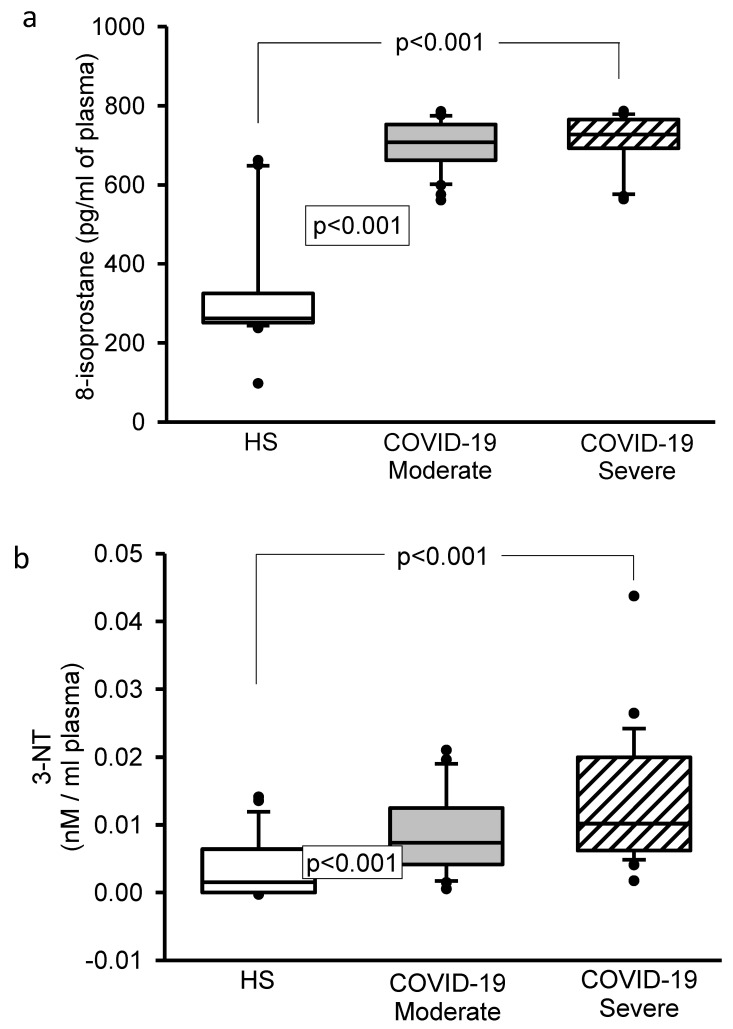
(**a**) The 8-isoprostane concentrations statistically increased in both the moderate and severe COVID-19 patients in comparison with HS. (**b**) 3-NT statistically increased in the moderate and severe COVID-19 patients in comparison to HS. Abbreviations: HS = healthy subjects, 3-NT = 3-nitrotyrosine.

**Figure 3:**
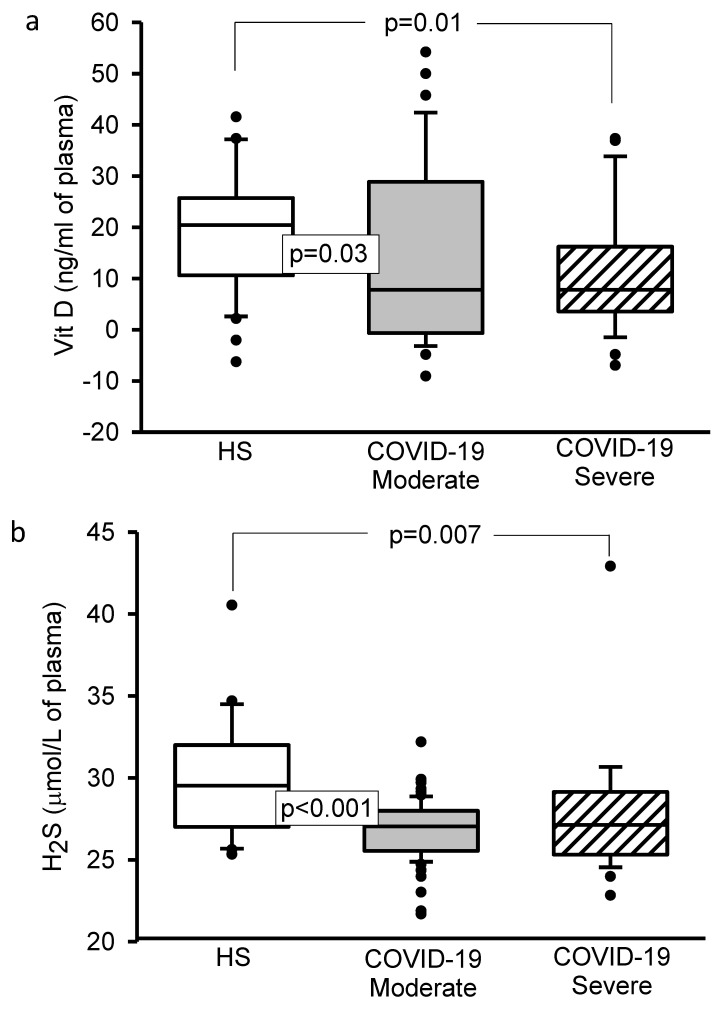
(**a**) Changes in the values of Vit D and (**b**) changes in the concentration of the H_2_S, there was a significant decrease in the moderate and severe COVID-19 patients in comparison with HS. Abbreviations: Vit D = Vitamin D, H_2_S = hydrogen sulfide, HS = healthy subjects.

The 8-isoprostane and 3-NT concentrations showed a significant increase (*p* < 0.001, Figure 2a,b). However, Vitamin D (*p* = 0.03 and *p* = 0.01, Figure 3a) and H_2_S (*p* < 0.001 and *p* = 0.007, Figure 3b) levels showed a decrease in moderate and severe COVID-19 patients in comparison to HS.

### 3.5. Oxidative Stress Markers

**Figure 4:**
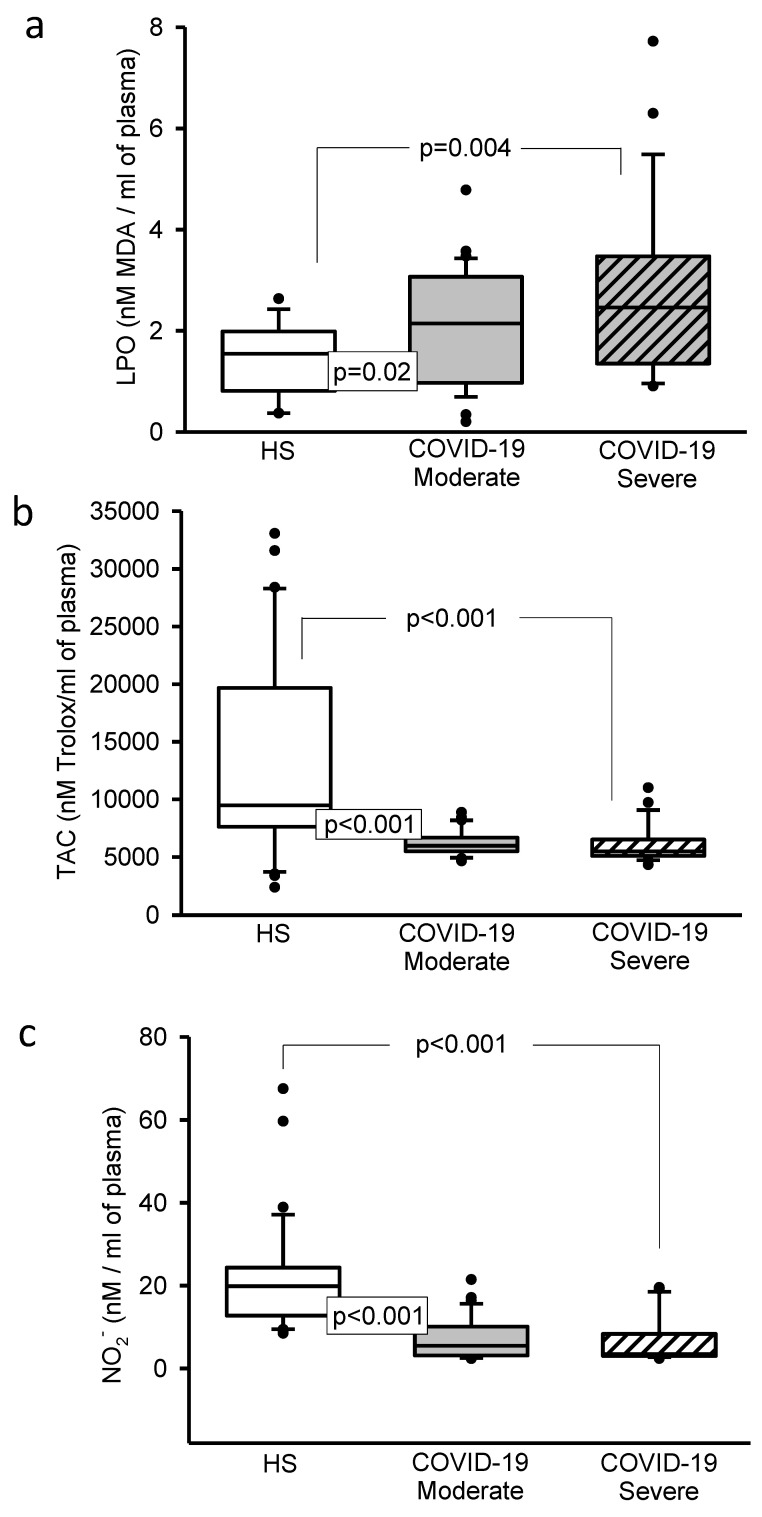
(**a**) The LPO index significantly increased in the moderate and severe COVID-19 patients in comparison with HS. (**b**) The TAC significantly decreased in the moderate and severe COVID-19 patients in comparison to HS. (**c**) The NO_2_^–^ concentration significantly decreased in the moderate and severe COVID-19 patients in comparison with HS. Abbreviations: LPO = Lipid peroxidation, TAC = Total antioxidant capacity, NO_2_^–^ = Nitrites.

**Figure 5:**
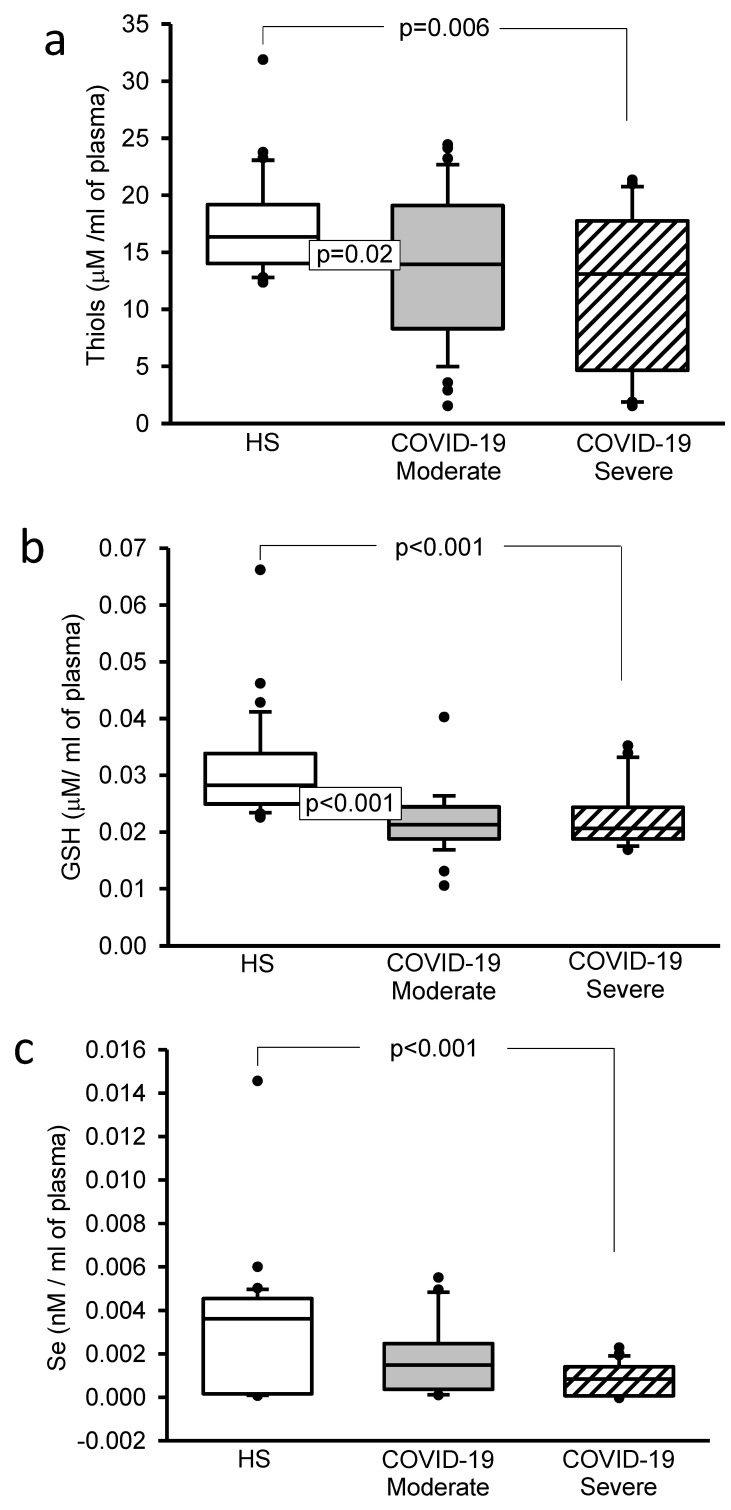
(**a**) The thiol concentrations significantly decreased in the moderate and severe COVID-19 patients in comparison to HS. (**b**) GSH concentration statistically decreased in the moderate and severe COVID-19 patients in comparison with HS. (**c**) The Se concentration significantly decreased in both the moderate and severe COVID-19 patients in comparison with HS. Abbreviations: HS = healthy subjects, GSH = glutathione, Se = selenium.

The LPO index showed a significant increase (*p* = 0.02 and *p* = 0.004, Figure 4a). However, the TAC (*p* < 0.001, Figure 4b), NO_2_^–^ (*p* < 0.001, Figure 4c), the thiols concentrations (*p* = 0.02 and *p* = 0.006, Figure 5a) and GSH levels (*p* < 0.001, Figure 5b) showed a decrease in moderate and severe COVID-19 patients in comparison with HS.

### 3.6. Selenium

The Selenium concentration only decreased in severe COVID-19 patients in comparison to HS (*p* < 0.001, Figure 5c).

## 4. Discussion

In the present study, the presence of anti-N protein, and anti-S protein antibodies of the SARS-CoV-2 virus was determined at the time of admission of the patients. In most of the patients, antibodies against these proteins were present at the time of admission and in the rest of them, the antibodies appeared a few days later. During the analytical validation of the ELISA to measure anti-N protein antibodies, it was found that up to 11% of subjects may not have measurable amounts of anti-N protein antibodies at the time of the manifestation of clinical symptoms. This was because these subjects are slow to produce antibodies and require a longer time to respond. However, between 4 and 10 days after the onset of the manifestations of clinical symptoms, patients already have measurable concentrations of anti-N protein antibodies. These results confirm the known fact that the measurement of anti-N protein antibodies is the most effective serological measurement for diagnosing the SARS-CoV-2 virus infection [15,22]. This is due to the fact that the N protein of the SARS-CoV-2 virus is a highly immunogenic protein that is overexpressed and released into the bloodstream simultaneously with the viral particles. Therefore, its measurement is a highly useful analytical tool for the epidemiological management of the pandemic [22]. The low positivity values for anti-S protein antibodies, both in the frequency of positive patients, and in the percentage of positivity, suggests that these patients had difficulty in producing neutralizing antibodies, necessary to counteract the infectious capacity of the SARS-CoV-2 virus [15,22]. For this reason, these subjects required hospitalization and were in need of specialized clinical management and even the use of artificial respirators.

On the other hand, several studies have shown that COVID-19 triggers a transient hyperglycemia and impairs pancreatic β-cell function. This has been associated with inflammation and the cytokine storm that may lead to IR. These changes contribute to a positive feedback cycle in the development and progression of hyperglycemia in COVID-19 patients. Hyperglycemia may also induce OS and glucolipotoxicity. However, in subjects with MS these changes are more aggressive, and comorbidities may lead to fatal outcomes [23]. Our results show that the hyperglycemia, hyperinsulinemia and IR were present in our series of 61 moderate and severe COVID-19 patients.

The association of the COVID-19 infection and altered glucose metabolism has previously been reported [24], and explained by the capacity of the virus to hijack mitochondrial function leading to an anaerobic function [5]. In this condition, there is an increase in lactate levels that provokes an increase in the dependency on glycolysis in hepatocytes, which is reflected in an increase in glucose and lactate levels in the blood. This state favors viral replication since the virus requires large amounts of energy for biosynthetical process [5]. Moreover, the increase in glucose levels elevates the pool of free fatty acids (FFA) which are necessary for the formation of viral membranes. It also increases nucleotides needed for RNA synthesis for viral replication [5].

As a consequence of glucose alterations, DM and MS patients have an increased risk of severe pneumonia by COVID-19 with fatal outcomes. This is reflected in a higher mortality in subjects with these comorbidities [25]. In addition, prolonged hyperglycemia could worsen the course of COVID-19 via glycation of pancreatic ACE2 and the transmembrane serine protease 2 (TMPRSS2), thus facilitating the SARS-CoV-2 binding and entrance to pancreatic β-cells [2]. However, SARS-CoV-2 may infect the pancreatic β-cells through other proteinases such as neuroplin-1 and transferrin receptor [26,27]. Studies in human islets infected in vitro and studies in postmortem autopsied tissue found a phenotypic alteration or trans-differentiation of the β-cells with decreased insulin and increased glucagon secretion that could lead to a fatal outcome [26,27]. However, these studies had great limitations such as the lack of a determination of circulating insulin concentrations in COVID-19 patients.

Our results showed that hyperglycemia and hyperinsulinemia were present in plasma of patients with and without comorbidities upon admission to the ICU. These results agree with those reported by Montefusco et al. who showed that in 551 patients hospitalized for COVID-19, the SARS-CoV-2 induces IR and disrupts β-cells function which can result in clinically evident hyperglycemia detectable even in the post-acute phase [28]. This suggests that both the hyperglycemia and hyperinsulinemia depend on the SARS-CoV-2 infection, the viral replication cycle and the viral load. As the disease progresses, there is an increase in glucose levels elicited by the demand of the virus for its replication, which favors a continuous overstimulation of the β-cell [24]. This results in an increase in insulin levels that eventually leads to depletion of insulin stores. This worsens hyperglycemia, and finally deteriorates β-cell function. In vitro studies have demonstrated that the hepatitis virus (HCV) infection of human β-cells also leads to hypersecretion of insulin in the initial stages of the infection and that it later causes a reduction in insulin in the β-cells [29]. Another study in pancreatic cells infected with HCV demonstrated that hyperinsulinemia and pancreatic β-cell hyperfunctionality is aimed to maintain glucose homeostasis [30]. Furthermore, hyperinsulinemia may disturb fibrinolysis by elevating the plasminogen activator inhibitor type 1 and increase thrombosis in COVID-19 patients [31].

An inadequate insulin secretion by decompensated β-cells may cause increasingly higher blood glucose levels that bathe the islet. This leads to a spectrum of consequences for the β-cell, including glucose desensitization, β-cell exhaustion, and eventually glucose toxicity [32]. Furthermore, this sustained response of the pancreas aimed towards maintaining glucose homeostasis, leads to the activation of pathways of apoptosis and trans-differentiation if it does not decrease the infection. These result, with time, in a decreased secretion of insulin by the β-cells and lower glucose-stimulated insulin secretion [33]. In addition, subjects without comorbidities may develop hyperglycemia after three days of the COVID-19 infection. This elevation in glucose levels can be reversed within 2 weeks. However, 10% of patients could develop DM later in time. This is important because these findings are not observed in other viral forms of pneumonia, suggesting the involvement of the pancreatic axis in the coronavirus infection [34]. Furthermore, subjects with DM show pre- and post-prandial hyperglycemia as well as diabetic ketoacidosis when infected by SARS-CoV-2 [34]. In our experimental series, out of 61 patients infected with SARS-CoV-2, 45 had comorbidities while 17 did not report comorbidities and, nevertheless, all presented alterations in glucose metabolism [34]. Hyperglycemia inactivates the glucose transporters (GLUT) which are normally triggered by insulin. In fibroblasts infected with the human cytomegalovirus, the IE72 trans activator of viral promoters decreases the mRNA of GLUT1 and increases the mRNA of GLUT-3, -4 and -8 from the early stage of infection that are 3 times more effective in transporting glucose. This increase is independent from the Akt-mediated metabolic pathway that depends on insulin concentrations. This suggests that the SARS-CoV-2 could probably have a similar metabolic pathway in glucose transport independent of hyperglycemia and hyperinsulinemia present in COVID-19 patients [35].

In addition, our results showed that the IR state in COVID-19 patients is present. In this sense, it has been described that binding of insulin to the insulin receptor or insulin-like growth factor 1 receptor (IGF1R) results in the autophosphorylation of insulin receptor substrate 1/2 (IRS1/2) at its tyrosine residues and in the subsequent activation of two main pathways, the phosphoinositide-3-kinase (PI3K-Akt) pathway and the mitogen activated protein kinase (MAPK) pathway. Conversely, serine phosphorylation of the IRS1/2 attenuates insulin signaling by decreasing insulin-stimulated tyrosine phosphorylation and this can produce IR [36]. A study using liver biopsy specimens obtained from non-diabetic HCV-infected patients showed that HCV impairs the insulin-stimulated tyrosine phosphorylation of hepatic IRS1, resulting in reduced PI3K-Akt activation without any alteration in the MAPK pathway [37]. Another study in a transgenic mouse model expressing the HCV genotype 1 core protein indicated that the core protein was responsible for inducing IR in the liver via suppression of tyrosine phosphorylation in IRS1, which is associated with DM [38].

During IR, the action of insulin at the cellular level is reduced in several tissues, which increases the secretion of this hormone by the pancreas. The HOMA-IR index is used as an indicator of IR [39]. IR is defined by an augmented production of glucose by the liver, reduced glucose uptake by muscle and elevated lipolysis. In this condition, muscles, adipose tissue and the liver do not respond to insulin. Moreover, IR is related to a range of risk factors for cardiovascular disease, including MS, hypertension, OS, dyslipidemia, inflammation and glucose intolerance [40,41]. Different studies have demonstrated that SARS-CoV-2 induces IR [42]. Our results show that COVID-19 patients presented an increase in the HOMA index. This result implies that during the progression of the infection, IR forces β-cells to elevate the synthesis of insulin trying to restore the normal blood glucose level [43]. Furthermore, IR leads to an increased pancreatic expression of the ACE2 receptors, increasing the binding affinity for the S protein of the SARS-CoV-2. Therefore, there is an increased vulnerability for the COVID-19 infection in subjects with IR [44].

The alterations in the glucose–insulin axis in COVID-19 patients with or without comorbidities, suggest that it is very important to control the glycemic state using different drugs such as the metformin, which improves IR and peripheral glucose uptake through the activation of AMP-dependent protein kinase [45]. Metformin also exerts pleiotropic effects through the AMP-independent pathway including anti-inflammatory and immunomodulatory effects and it also inhibits the synthesis and release of CRP, IL-1β-induced IL-6, and ferritin from macrophages, endothelial cells, smooth muscle vascular cells and hepatocytes [46]. Metformin also reduces the binding of SARS-CoV-2 to ACE2 by inducing functional changes in the transmembrane enzyme by AMP-dependent phosphorylation [47].

Hyperglycemia, hyperinsulinemia and IR contribute to increase ROS that attack free fatty acid (FFA) or esterified arachidonic acid in the phospholipids of the membrane and constitute conditions that are frequently found in COVID-19 and in patients with MS. Hyperglycemia, hyperinsulinemia and IR increase the rate of lipolysis which is accompanied with liberation of FFA to the circulation. An increased fatty acid (FA) metabolism is essential for the formation of the viral membranes including those of SARS-CoV-2 [48]. Furthermore, the increase in FFA and the alteration in FA metabolism increase gluconeogenesis, reduce β-cells mass and provide substrates for the synthesis of triglycerides that are elevated in COVID-19 patients [48]. The attack of the membrane phospholipids and arachidonic acid by ROS result in the formation of isoprostanes that are molecules that are considered as markers of LPO and oxidative injury. In this sense, 8-isoprostane has been considered an ideal marker for pathophysiology such as bronchoalveolar lavage fluid of patients with interstitial lung disease [49]. Our results show that 8-isoprostanes and IL-6 were elevated in both moderate and severe COVID-19 patients. A study in rats with EPOC also showed an increase in 8-isoprostanes and IL-6 in lung lysates associated with ACE2 overexpression and another study demonstrated an increase in urinary 8-isoprostanes levels in patients with chronic obstructive pulmonary disease [50,51]. This suggests that the 8-isoprostanes are associated in pneumonia by SASR-CoV-2 and comorbidities present in MS and that their synthesis is a consequence of the alteration of the FA metabolism and the oxidative background. These alterations contribute to ACE2 over-expression that facilitates the entrance of the virus into the host cells. However, more investigations are necessary to verify this hypothesis. In addition, the alterations in the glucose–insulin axis and FA metabolism [48] may lead to an increase in OS, increased inflammation, interleukin storm, and decreased mobilization of leukocytes, phagocytic activity, TAC and impairment of endothelial function [52]. In this sense, it has been reported that the TAC levels were considerably lower in serum of mild and severe COVID-19 patients in comparison with control subjects [53]. Our results showed alterations in some markers of OS such as LPO, TAC, thiols, 8-isoprotanes, GSH, 3-NT and NO_2_^–^ concentrations. In ARDS and COPD associated with COVID-19, the hypoxic conditions result in inflammation that may lead to OS. Increased OS leads, as a consequence, to a decrease in nitric oxide (NO) release mainly because ROS interfere with the activity of the NO synthases and cause an inadequate production of tetrahydrobiopterin [54]. Optimal concentrations of O_2_ are required for the synthesis of NO by the nitric oxide synthases.

Changes in the activity of these synthases are reflected in a decrease in their metabolites including NO_2_^–^. This metabolite was decreased in moderate and severe patients in our study. Furthermore, the newly synthetized NO might be oxidized by ROS to peroxinitrite (ONOO^–^) which is the most aggressive RNS and may contribute to the inflammatory process associated with the interleukin storm in the COVID-19 patients. Low levels of NO also induce proliferation of vascular smooth muscle cells, platelet aggregation, elevated pro-inflammatory cytokines such as IL-6, chemokine expression, low-density lipoprotein oxidation, and expression of vascular cell adhesion molecule-1, stimulation of thrombolysis and monocyte chemotactic protein-1 through the inhibition of the NF-κB signaling pathway. Moreover, decreased NO following an oxidative burst can stimulate the production of metalloproteinases-2 and -9 that contribute to pulmonary damage [55]. Hypoxia also increases the presence of protons, in a similar way as in a neutral or acidic pH, compromising the stability of ONOO^−^ and decomposing peroxynitrous acid to form nitrates (NO_3_^−^) and NO_2_^−^ [54]. ONOO^−^ causes the irreversible process of nitration of tyrosines in proteins, it oxidizes the thiol groups in them [56] and it also lowers the H_2_S concentration. In this sense, H_2_S is a potent gasotransmitter that is decreased in COVID-19 patients [57]. Recent studies provide evidence of the beneficial role of H_2_S in COVID-19 patients. In 10 patients with severe COVID-19 intravenous N-acetylcysteine a potential H_2_S releaser decreased the inflammation [58]. H_2_S is able to positively modulate concentrations of cytokines by reducing pro-inflammatory IL-6 and TNF-α. This suggests that the decrease in the H_2_S levels contributes in part to a pro-inflammatory state in SARS-CoV-2 infection. In addition, moderate or high IL-6 levels have been associated with the decrease in the cysteine and taurine concentration which are essential amino acids for H_2_S synthesis [59]. Our results show that 3-NT in plasma proteins was increased both in moderate and severe patients. This result suggests that COVID-19 courses with nitrosative stress (NSS) [5,60], which is associated with ferroptosis [5].

Ferroptosis results from mitochondrial sequestration by the viruses and contributes to an oxidative environment by increasing the Fenton–Haber–Weiss reaction which results in OS and NSS production [5], and in the ferroptosis present in COVID-19 patients [61]. The NSS and OS damage disulphide bonds (thiols), which are necessary to stabilize the architecture of proteins. Viruses and pathogens rely on the proper redox state for their –S–S– bonds or sulfhydryl groups that are needed for their entrance to the host cells. The S1 subunit is responsible for receptor binding and it contains the receptor-binding domain which contains disulphide bridges that are indispensable for the union with the ACE2. Moreover, the ACE2 receptor and the antioxidants enzymes also contain disulphide bridges, and therefore, dynamic disulphide bridge homeostasis is very important for both viral replication and for the antioxidant defense in the patient [3]. Our results show that the level of thiols in plasma from patients with COVID-19 was decreased in moderate and severe cases. This result is similar to the one found in another study where it was demonstrated that COVID-19 patients’ course with low levels of thiols and that their level can even be a marker that reflects the gradual increase in the severity of the infection [62]. This result is very important because different investigations have reported that the treatment with agents capable of elevating the reducing thiol groups such as GSH, allicin, garlic and N-acetylcysteine, can restore the homeostasis of thiols and decrease the degree of viral infection increasing the TAC and decreasing the LPO in COVID-19 patients [63].

On another hand, the low levels of H_2_S favor a decrease in the thiol concentration because this gasotransmitter reduces the –S–S– in the proteins and enzymes and increases the expression of TMPRSS2 which facilities the SARS-CoV-2 entrance into cells [64]. Low levels of H_2_S have been reported in ARDS and COPD associated with the SARS-CoV-2 infection [65]. In this sense, exogenously applied H_2_S donors such as NAC protect from lung damage, including that produced by ARDS, COPD, ALI, asthma pulmonary fibrosis and hypoxia-induced pulmonary hypertension in animal models and in humans. This therapy has therefore been proposed as an alternative strategy in this pandemic [66].

**Figure 6:**
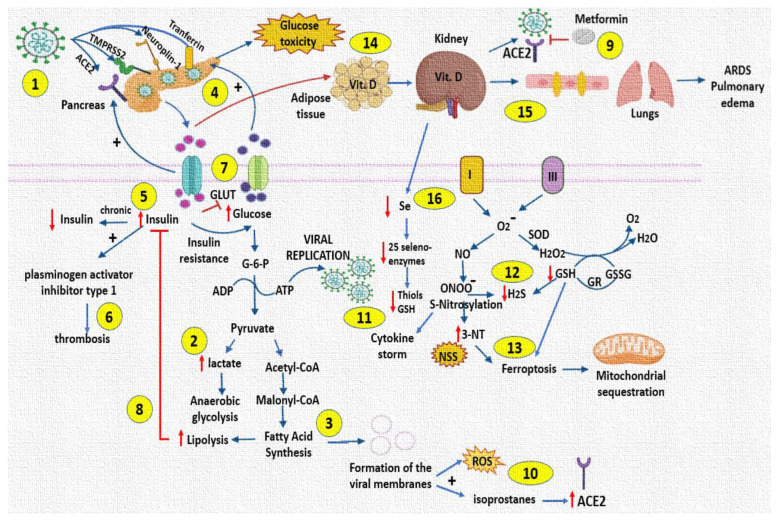
Alteration of the glucose–insulin axis by SARS-CoV-2, and the impact on some antioxidant markers in patients. (**1**) Entry of COVID-19 through various receptors in the pancreas. (**2**) COVID-19 increases lactate levels, favoring viral replication. (**3**) Increased free fatty acids are used for viral membrane formation. (**4**) Increased glucose overstimulates pancreatic β-cells and subsequently impairs their function. (**5**) Chronically increased insulin concentrations will lead to depletion of insulin reserves. (**6**) Hyperinsulinemia may favor the development of thrombosis. (**7**) Hyperglycemia inactivates GLUT 1 but SARS-CoV-2 could increase -3, -4 and -8, transporters. (**8**) Increased lipolysis blocks the response to insulin by the liver, adipose tissue and muscles. (**9**) Metformin prevents the interaction of the ACE2 receptor and COVID-19. (**10**) Increased free fatty acid synthesis favors the generation of 8-isoprostanes, which favor ACE2 receptor overexpression. (**11**) ONOO^–^ favors interleukin storm. (**12**) ONOO^–^ decreases H_2_S concentration. (**13**) Nitrosative stress and depletion of GSH concentrations is associated with ferroptosis and mitochondrial sequestration by the virus. (**14**) Under conditions of hyperinsulinemia, Vitamin D is sequestered in adipose tissue and inactivated in the kidney, which favors the interaction of virus S protein with the ACE2 receptor. (**15**) Vitamin D deficiency generates ruptures in the gap junctions of lung endothelial cells. (16) Vitamin D deficiency is associated with Se deficiency which affects selenoenzymes. Abbreviations: ARDS = acute respiratory distress syndrome, H_2_O_2_ = hydrogen peroxide, H_2_S = sulfhydryl acid, G-6-P = glucose 6 phosphate, GR = glutathione reductase, GSH = glutathione, GSSG = oxidized glutathione, NO = nitric oxide, NSS = nitrosative stress, O_2_^–^ = superoxide, ONOO^–^ = peroxynitrite, ROS = reactive oxygen species, SOD = Superoxide dismutase.

Moreover, levels of Vitamin D are low in diseases that course with hyperinsulinemia such as COVID-19 and in the pathologies that comprise the MS [67,68]. Vitamin D exerts its effects by binding to a nuclear Vitamin D receptor, which is expressed in various immune cells, with particularly high levels in dendritic cells, macrophages, T and B lymphocytes that are increased when the innate immune response is elicited [69]. During hyperinsulinaemic states, Vitamin D3 is sequestered into adipocytes, and inactivated in the kidney [42]. Our results show that Vitamin D levels were decreased in moderate and severe COVID-19 patients. This suggests that hyperinsulinemia may decrease the concentration of this vitamin having an unfavorable impact upon the innate immune response. Moreover, this deficiency may increase the interaction of the S protein with ACE2, because its low levels favor an increase in the TMPRSS2 protease which is essential for the entrance of the virus [70]. Vitamin D deficiency also decreases innate cellular immunity by lowering the expression of defensins which maintain the gap junctions in the endothelial cells of the lung. Rupture of these junctions is caused by the SARS-CoV-2 leading to ARDS and pulmonary edema [71]. High concentrations of Vitamin D may also have benefic effects such as the induction of the vasorelaxant ACE2/Ang-(1-7)/Mas receptor axis, which protects against acute lung injury and ARDS [72]. In addition, low levels of Vitamin D are associated with Se deficiency and are related to COVID-19 severity, old age, obesity, diabetes and dyslipidemia. However, in COVID-19 patients without comorbidities, there is also a decrease in Vitamin D and Se in comparison with the healthy subjects [68]. The participation of Se is also important in COVID-19 because it has synergic effects with Vitamin D and E acting as antioxidants. Furthermore, Se is indispensable for the presence of 25 seleno-enzymes such glutathione peroxidase, thioredoxin reductases (TrxR), deiodinases and methionine sulfoxide reductases which play an important role in maintaining the redox homeostasis [73]. Our results show that the concentrations of Se and GSH were decreased in severe COVID-19 patients. This suggests that the redox homeostasis which is dependent on the 25 seleno-enzymes is altered and this condition is associated with severe COVID-19. Moreover, this impacts on the presence of low thiols GSH and H_2_S concentrations in plasma, which contribute to an increase in OS and NSS present in these patients. This is reflected in the rise of the LPO index accompanied with depletion of the TAC. In this sense, Se deficiency in mice was associated with enhanced virulence of enteroviruses and the development of myocardial lesions [73]. The homeostasis of thiols and GSH depend on TrxR and glutathione reductase which are seleno-enzymes. Low levels of the GSH in COVID-19 patients are associated with ferroptosis and with a down-regulation of GPX4 and TrxR [5,61]. A deficiency in GSH is linked to HIV progression and poor survival of HIV-infected individuals [74], and high concentrations of GSH in blood decrease the virulence of the infection by dengue and chikungunya [75]. Our results suggest that a reduction in the GSH concentration may contribute to an increase in OS and NSS and favor the decrease and increase, respectively, in the TAC and LPO in COVID-19 patients. Figure 6 summarizes the alterations in the glucose–insulin axis by SARS-CoV-2.

## 5. Conclusions

The results suggest that infection with SARS-CoV-2 in patients with and without comorbidities results in alterations in the glucose–insulin axis which leads to hyperglycemia, hyperinsulinemia and IR. These alterations increase OS and NSS that is reflected in increases or decreases in some oxidative markers in plasma with major impacts or fatal consequences in patients that course with MS. Moreover, subjects without comorbidities could have long-term alterations in the redox homeostasis after infection.

### 5.1. Study Limitations

One of the limitations of this study is that we could not study SARS-CoV-2-infected subjects with mild symptoms or asymptomatic COVID-19. The center in which this work was carried out was a reference center and received patients with moderate to severe symptoms sent from other hospitals. Another limitation is that in the evaluation of certain parameters such as D-Dimer, Ferritin, N/L index, there is a tendency to an elevation in moderate and critical patients; however, this cannot be defined in a concrete way because a larger sample size would be required for this specific evaluation. However, it is known that these parameters increase, and our results show this same tendency. It is important to state that the sample for this study was adjusted for the specific question of the investigation. One of the limitations of this study is also that we were not able to measure markers with thromboelastography to define the co-participation of a hyperglycemic state with changes in PAI-1 and fibrinolysis. It is currently known that a subset of patients with COVID-19 have a higher risk of bleeding, and high levels of tissue-type plasminogen activator (tPA) and plasminogen activator inhibitor 1 (PAI- 1) have been associated with a worse lung function. An elevated tPA is independently correlated with mortality. The levels of any of these molecules can increase independently of the other; however, a change in one can have consequences on another. The interaction of plasminogen activators, both tissue-type (tPA) and urokinase-type, and their main inhibitor, PAI-1, play a key role in the regulation of fibrinolytic activity. Impaired fibrinolysis has been suggested in COVID-19 patients, which could further increase thrombotic risk. This has been evidenced by markedly reduced clot lysis at 30 min by thromboelastography in critically ill COVID-19 patients [76]. Another limitation of the study is that the oxidative markers can be altered by the age, gender and pathological comorbidities which was not contemplated in this work but would be interesting to address in a future investigation.

### 5.2. Perspectives

According to the results of our study, the use of a combined therapy with antioxidants such as NAC and metformin could attenuate SARS-CoV-2 infection in patients with COVID-19, as has already been shown in various studies [11].

## Figures and Tables

**Table 1:** Demographic characteristics at admission of patients infected with COVID-19.

	Total *n* = 61 (100) Number and Percentage	Moderate *n* = 34 (56) Number and Percentage	Severe *n* = 27 (44) Number and Percentage	*p*
Women	17 (28)	11 (32)	6 (22)	NS
Men	44 (72)	23 (68)	21 (78)	NS
Age	56 ± 13	54 ± 12	59 ± 14	NS
BMI (kg/m^2^)	29 ± 4	29 ± 4	29 ± 4	NS
Temperature (°C)	36.6 ± 0.46	36.5 ± 0.43	36.7 ± 0.49	NS
Laboratory at the admission Median (Min–Max) range				
PAO_2_ (58.5–67.1 mmHg)	66.9 (34–223)	67 (34–223)	66.4 (62–93)	NS
PCO_2_ (30.4–40 mmHg)	31.7 (12.2–81.2)	32 (12.2–81.2)	31.6 (22–71)	NS
PAO_2_/FIO_2_ (>164)	128 (26.8–299)	145 (26.8–281)	113 (30–299)	NS
SpO_2_/FIO_2_ (>300)	138 (50–280)	157 (88–240)	128 (50–280)	0.007
Urea (<40 mg/dL)	16 (5.6–106.7)	29.2 (16–60)	30 (13–224)	NS
Creatinine (mg/dL)	0.90 (0.5–5.3)	1 (0.5–2.5)	0.8 (0.5–5.3)	NS
Ureic Nitrogen (7–25 mg/L)	16 (5.6–196-7)	15.7 (7.5–36)	17 (5.6–106.7)	NS
TC (<200 mg/dL)	136 (69–217)	140 (69–217)	132 (86–190)	NS
HDL (mg/dL)	31 (14–60)	32 (14–60)	31 (14–45)	NS
LDL (mg/dL)	70 (28–140)	64 (35–135)	79 (28–140)	NS
DHL (mg/dL)	253 (124–515)	233 (128–412)	255 (124–515)	NS
TB (mg/dL)	0.60 (0.12–4.10)	0.50 (0.12–1.3)	0.70 (0.33–4.10)	0.02
DB (mg/dL)	0.20 (0.10–1.20)	0.20 (0.10–1.2)	0.20 (0.10–0.8)	NS
TG (<150 mg/dL)	133 (62–726)	137 (62–726)	133 (77–328)	NS
Leukocytes (3.5–10.3 × 10^3/µL^)	8.8 (2–25)	8.2 (2–14)	11.5 (3–25)	0.003
Lymphocytes (0.99–3.2 × 10^3/µL^)	0.8 (0.14–9.6)	0.92 (0.42–9.6)	0.69 (0.14–8.2)	0.01
Platelets (150,000–500,000 × 10^3/µL^)	244 (16–576)	226 (16–576)	254 (122–412)	0.057
Ferritin (11–307 ng/mL)	541 (147–2592)	513 (147–2100)	592 (175–2592)	NS
IL-6 (pg/mL9)	67 (7.8–638.5)	30.2 (7.8–304)	94 (7.8–639)	0.003
Index N/L	11.5 (1–89)	10 (3–89)	13 (1–83)	NS
D-Dimer (0–24 µg/mL)	700 (136–16440)	615 (136–5130)	810 (210–16,640)	0.08
CRP (1–3 mg/L)	146 (20–2450)	280 (20–1380)	146 (32–2450)	NS
Comorbidities (%)				
DM	6 (10)	4 (7)	2 (3)	NS
SAH	5 (8)	3 (5)	2 (3)	NS
Dyslipidemia	11 (18)	8 (13)	3 (5)	NS
DM + Dyslipidemia	5 (8)	2 (3)	3 (5)	NS
DM + SAH	5 (8)	2 (3)	3 (5)	NS
SAH + Dyslipidemia	3 (5)	0	2 (5)	NS
SM	9 (15)	6 (10)	3 (5)	NS
Healthy Subjects without comorbidities	17 (28)	8 (13)	9 (15)	NS
Normal weight	13 (21)	6 (18)	7 (26)	NS
Overweight	24 (39)	17 (50)	7 (26)	0.06
Obesity	24 (39)	11 (33)	13 (48)	NS
COPD	1 (1.6)	0	1 (4)	NS
ECKD	2 (3.3)	0	2 (8)	NS
Norepinephrine	18 (29)	1 (3)	17 (63)	0.0001
Enteral nutrition	26 (43)	22 (65)	4 (15)	0.001
Deaths	1 (1.6)	0	1 (4)	NS

Abbreviations: BMI = Body mass index, HR = Heart rate, MAP = Mean arterial pressure, HDL = High-density lipoproteins, LDL = Low-density lipoproteins, TB = Total bilirubin, DB = Direct bilirubin, IL = Interleukin, N/L = neutrophil&/lymphocyte, DM = Diabetes mellitus, SAH = Systemic arterial hypertension, CD = Cardiovascular disease, COPD = Chronic obstructive pulmonary disease, ECKD = End-stage chronic kidney disease. FiO_2_ = inspired fraction of oxygen, PAO_2_ = Blood pressure oxygen, PCO_2_ = Partial pressure carbon dioxide, SpO_2_ = Arterial oxygen saturation, ECKD = End-stage chronic kidney disease, TC = Total cholesterol, TG = Triglycerides, MS = Metabolic syndrome.

## Data Availability

The datasets generated and analyzed during the current study are available from the corresponding author on reasonable request.
